# Zebrafish Fins as a Model System for Skeletal Human Studies

**DOI:** 10.1100/tsw.2007.190

**Published:** 2007-07-03

**Authors:** Manuel Marí-Beffa, Jesús A. Santamaría, Carmen Murciano, Leonor Santos-Ruiz, José A. Andrades, Enrique Guerado, José Becerra

**Affiliations:** ^1^ Department of Cell Biology, Genetics and Physiology, Faculty of Science, University of Málaga, Louis Pasteur Avenue, 29071-Málaga, Spain; ^2^ Hospital Costa del Sol, Division of Orthopaedic Surgery and Traumatology, 29600-Marbella, Spain

**Keywords:** bone, regeneration, vertebrates, actinopterygian fishes, mammals, humans, cell therapy, mesenchymal stem cells, bone morphogenetic proteins, transforming growth factor beta, sonic hedgehog, intramembranous ossification, endochondral ossification, ray dermal bone, lepidotrichia, extracellular matrix, collagen, osteocyte, scleroblast, bone repair, bone regeneration, regenerative medicine, enamel, dentine

## Abstract

Recent studies on the morphogenesis of the fins of *Danio rerio* (zebrafish) during development and regeneration suggest that a number of inductive signals involved in the process are similar to some of those that affect bone and cartilage differentiation in mammals and humans. Akimenko et al. (2002) has shown that bone morphogenetic protein-2b (BMP2b) is involved in the induction of dermal bone differentiation during fin regeneration. Many other groups have also shown that molecules from the transforming growth factor-beta superfamily (TGFβ), including BMP2, are effective in promoting chondrogenesis and osteogenesis *in vivo* in higher vertebrates, including humans. In the present study, we review the state of the art of this topic by a comparative analysis of skeletal tissue development, regeneration and renewal processes in tetrapods, and fin regeneration in fishes. A general conclusion of this study states that lepidotrichia is a special skeletal tissue different to cartilage, bone, enamel, or dentine in fishes, according to its extracellular matrix (ECM) composition. However, the empirical analysis of inducing signals of skeletal tissues in fishes and tetrapods suggests that lepidotrichia is different to any responding features with main skeletal tissues. A number of new inductive molecules are arising from fin development and regeneration studies that might establish an empirical basis for further molecular approaches to mammal skeletal tissues differentiation. Despite the tissue dissimilarity, this empirical evidence might finally lead to clinical applications to skeletal disorders in humans.

## INTRODUCTION

One of the main goals of evo-devo synthesis is the search for genetic and molecular embryological synapomorphies to support systematics at a molecular level. For this purpose, some authors have attempted to describe the phylogenetic relationship among the main types of skeletal tissues[1,2]. There are four main types of skeletal tissues: bone, cartilage, enamel, and dentine[3]. In order to extend this analysis to all skeletal tissues, we will focus our analysis on the actinopterygian fins skeleton, using zebrafish as a model system. Actinopterygian ray-fins are composed of three different types of skeletal structures: basal endochondral bone, lepidotrichia (the distal ray dermal bone skeleton [Fig. 1.A]), and actinotrichia. Previous papers have considered that lepidotrichia is similar to enamel, but not to dentine[4]. Further studies suggest that dental tissues and dermal skeletal tissues are distant in evolutionary terms[2]. Other studies propose that lepidotrichia is similar to cartilage, based on its collagen fibrils[5] and collagen-proteoglycan interactions[6]. Recent molecular studies[7,8] finally suggest that lepidotrichia is similar to both cartilage and bone. We discuss these possibilities in search of general arguments for a comparative molecular histology of skeletal tissues in fishes.

We shall review the composition and architecture of the extracellular matrix (ECM) of the lepidotrichia of zebrafish fins[7] and compare it with that of cartilage, bone[8], enamel, and dentine in fishes[9,10]. This study will be extended to how different skeletal tissues respond under a variety of experimental conditions[11,12].

Amphibians, chicks, and several mammals are model systems used in the search for molecular mechanisms that control skeletal tissues formation[13,14,15,16]. Nowadays, clinical treatments of skeletal disorders are based on experiments carried out in these species, especially in mammals. Cells of the chondrogenic and osteogenic lineages can be isolated and following *in vitro* manipulation of the mesenchymal stem cells (MSCs), these cells can be used for autologous skeletal tissue repair, using an *ex vivo* technique in human patients[17] or in the experimental animal models[18,19,20,21,22]. The results are similar to those obtained after experimental gene therapies for cartilage and bone disorders in mammals[23,24,25]. The zebrafish has previously been proposed as a model system in preclinical studies of skeletal tissue diseases[26,27]. We shall review empirical molecular and cellular mechanisms that induce the formation of lepidotrichia vs. other skeletal tissues in vertebrates. All the arguments presented in this paper support the notion that, although lepidotrichia is a special type of skeletal tissue, fin ray regeneration in zebrafish could also be useful for preclinical studies of skeletal tissue disorders.

## ALTHOUGH RAYS IN ACTINOPTERYGIAN FISHES DISAPPEARED THROUGH EVOLUTION IN VERTEBRATES, CERTAIN GENERAL MORPHOLOGICAL FEATURES OF LEPIDOTRICHIA ARE SOMEHOW SIMILAR TO SOME HIGHER VERTEBRATE SKELETAL TISSUES

Fin types, fin general morphology (Fig. 1A), and number of fin rays[29] are characteristics used in actinopterygian systematics. Rhipidistian are crossopterygian fishes with enlarged basal bones at the expense of a reduction in distal dermal rays[30]. These fossil devonian fishes are at the base of amphibians and the rest of vertebrates with tetrapod limbs. Among fossil fishes, terrestrial colonization by vertebrates was preceded by the transformation of pectoral and pelvic fins into tetrapod limbs. This process occurred with the loss of dermal fin rays, overgrowth of basal bones, and formation of digits (Fig. 1B)[3]. Dermal fin rays of actinopterygian fishes are then nonhomologous to tetrapod limbs.

Sections of fin rays reveal a characteristic lepidotrichia tissue that resembles a parenthesis surrounding the connective tissue containing blood vessels and nerves (Fig. 1C from *Carassius auratus*), and a covering multistratified epidermis. Lepidotrichia can be either cellular or acellular. Lepidotrichia cellular bone shows many cells immersed in a calcified and laminar ECM. In general, this ECM is synthesized by peripheral scleroblasts that build the matrix and may become trapped in it. A thin layer of an osteoid-like matrix can also be observed near the scleroblast layer[31].

In general, the other skeletal tissues (bones, cartilage, enamel, and dentine) are also constituted by particular cell types and characteristic composition of ECM molecules. As an example, we show the histological appearance of membrane and endochondral bone and fibrous cartilage. Details on other skeletal types can be found in Hall[3]. Intramembranous ossified bones (i.e., bones of the skull) show a

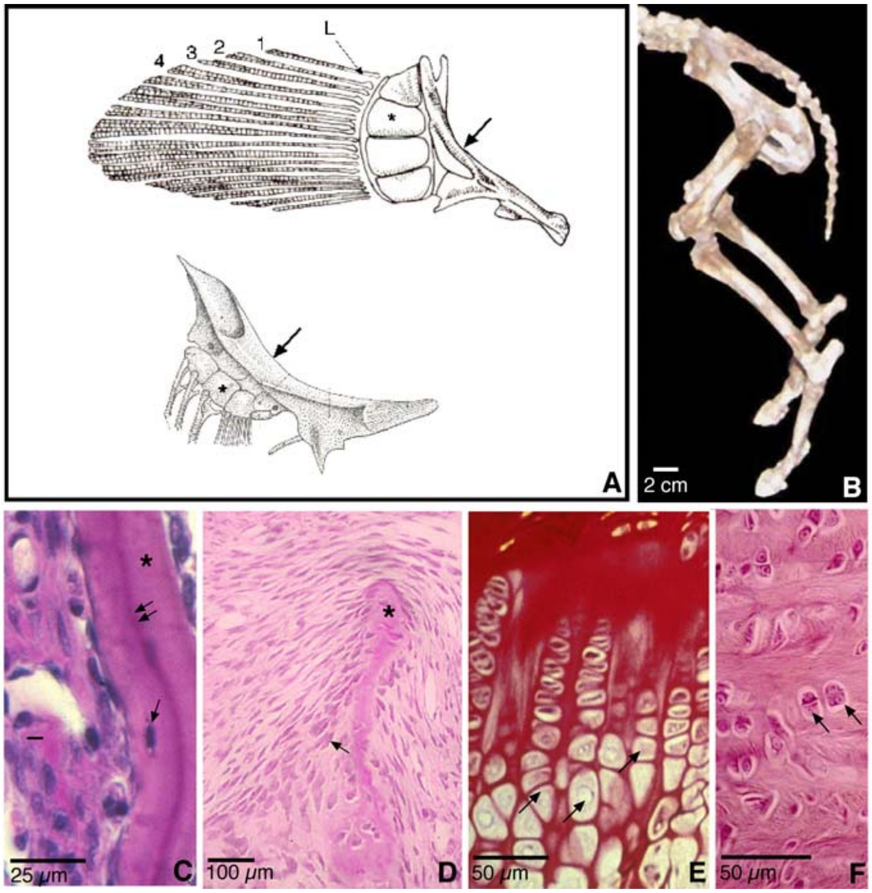

**FIGURE 1.** (A) Schematic representation of a pectoral fin skeleton of a teleost (*Gobius*). Observe how the dermal rays, mostly formed by lepidotrichia (L) (only numbered from 1 to 4), are organized distal to the cleithrum (arrow) and hypercoracoid bones (asterisk). Below this general scheme, observe a detail of the scapular girdle of *Trigla* (differentiating by endochondral ossification and similar to the one shown in *Gobius*) (adapted from Grassé[28]). In rhipidistian fishes, the dermal rays start disappearing and the basal bones overgrow as a general vertebrate evolutionary trend until the rays are completely absent in the limb as in actual birds or mammals. (B) The posterior part of *Ovis aries* (sheep) skeleton shows how the basal bones overgrow, articulate, and bifurcate forming the digits (bottom), reaching large proportions as compared to actinopterygian fishes. (C) The dermal bone of the rays is also segmented and bifurcated. In cross-section, obtained from a caudal fin of a *C. auratus* specimen, the dermal bone (lepidotrichia) is a parenthesis-like structure (asterisk) — in this picture, only one element of the parenthesis is shown — that surrounds a loose connective tissue with fibroblasts and blood vessels immersed in the tissue. In the case of *C. auratus*, this is a cellular bone as cells can be observed inside the lepidotrichia (arrow). The lepidotrichia is divided into two parts, one internal and one external, separated by a specially glycosilated ECM (double arrows). (D) Detail of a bone growing by intramembranous ossification in humans. Observe a central condensation (asterisk) and various strata of osteocytes (arrow) providing a general laminar ordering. (E) Detail of a bone growing by endochondral ossification in a rat (*Ratus ratus*). Observe the bone matrix (in red) with osteocytes. Long bones grow at the epiphyseal plate by the hypertrophy of a template of cartilage (arrows) and its substitution by migrating osteoblasts. (F) Fibrous cartilage. Observe chondrocytes (arrows) and fibrillar ECM surrounding them. Magnifications are shown by bars.

similar structural pattern: cells (osteocytes) immersed in a calcified and laminar ECM deposited by peripheral osteoblasts in a subepidermal location (Fig. 1D). By contrast, the general morphology of endochondral bones is more complex and involves a stereotyped order of cells at different maturation and differentiation stages at the growing sites (i.e., the epiphyseal plate of the long bones) (Fig. 1E). Chondrocytes proliferate, become prehypertrophic, then hypertrophic, and provide a cartilage template for migrating progenitor cells that differentiate into osteoblasts that finally synthesize a calcified ECM, the bone matrix, with immersed osteocytes. Between trabecular bone, bone marrow (BM) is formed that contains hematopoietic and mesengenic cell lineages[32]. Fibrous cartilage is composed of an ECM with thick bundles of collagen fibrils that surround chondrocytes. Mineralization and ground substance masking collagen is absent except for regions neighboring chondrocytes (Fig. 1F). Similar processes have been described to occur in developing osteichthyan fishes, such as zebrafish[3].

The cellular types of the other main skeletal tissues, enamel[33] and dentine, are the ameloblasts and odontoblasts, respectively. Enamel and dentine are also highly mineralized tissues[3]. The composition and formation of fish dentine and enamel have been recently described and compared with the corresponding tissues in tetrapods[2,10,34]. Histologically, lepidotrichia is then similar to the main skeletal tissues and is described as a dermal bone[35]

## MSCS CAN BE INDUCED TO DIFFERENTIATE INTO ANY SKELETAL TISSUE BOTH *IN VITRO* AND *IN VIVO*

Mesenchymal cells obtained from the BM maintain the capacity of differentiating either in chondroblasts or in osteoblasts in culture[36]. These cells may be reintroduced into vertebrates with bone disorders and repair mechanisms activated *in vivo*. All main skeletal tissues formation can be ectopically induced by this method[37,38].

In general, the development of skeletal tissues occurs by stereotyped developmental patterns. Mesenchymal cell migration, condensation, alignment, secretion of a specific composition of ECM molecules, and cell differentiation may occur. Appositional growth and mineralization are also typical of these tissues. Differentiation of cells competent to inducing signals also occurs in a sterotyped manner. In this study, competent cells are osteoblasts, differentiating into osteocytes (bone); chondroblasts, differentiating into chondrocytes (cartilage); preameloblasts, differentiating into ameloblasts (enamel); preodontoblasts, differentiating into odontoblasts (dentine); and scleroblasts precursor cells (SPCs) that differentiate into scleroblasts (lepidotrichia). Except for SPCs, all these precursor cells may arise from MSCs *ex vivo* induction[20,37,39,40,41]. In general, a microenvironment that controls stem cell activity, the Niche, has been described[42].

Zebrafish cartilage, bone, and even tooth development, which occurs through a continuous eruption and replacement of teeth at the pharyngeal arches, has been described[3,43]. However, the clear existence of MSCs in fishes has not been demonstrated yet[44]. In relation to this topic, Nechiporuk and Keating[45] suggest the absence of stem cells in zebrafish fins during regeneration (see below) and support the notion that all cells are equally competent to dedifferentiate following fin ablation.

## FIN REGENERATION AFTER AMPUTATION RESTORES MORPHOLOGY AND FUNCTION; THIS PROCESS IS SIMILAR TO TETRAPOD LIMB REGENERATION

The tetrapod limb is a classical system to study regeneration. Hundreds of experiments have been performed using this system. However, the fin is also capable of regeneration and represents a simpler regenerating system than the tetrapod limb. Only tissues supported by lepidotrichia and actinotrichia can regenerate. This process includes epidermal cell migration, mesenchymal cell dedifferentiation and migration, and blastema formation and outgrowth[46] similar to tetrapod limb regeneration[47]. Both limb and fin regeneration also require tissue interactions[48,49,50,51,52]. However, while in the regenerating fin, histological analysis[53] has shown that a number of blastemal cells that adjoin the basal epidermis differentiate into scleroblasts that synthesize the lepidotrichia[35,53], therefore suggesting the participation of epidermal-blastema interactions for bone regeneration, cartilaginous condensation occurs away from the epidermis during tetrapod limb regeneration[47]. Recent studies further suggest that fetal mammals may also partially regenerate their limbs using mechanisms similar to lower vertebrate limbs[54].

Besides endochondral bone and lepidotrichia, other skeletal tissues may also regenerate. For example, deer antler regeneration has been proposed as a model system for intramembranous bone regeneration[13,55]. A complete comparative tissue and molecular analysis of the regeneration of the different types of skeletal tissues will require further investigations.

## BASIS FOR A COMPARATIVE MOLECULAR ANALYSIS OF SKELETAL TISSUES

Theoretically, tissue ontogeny, renewal, and regeneration of skeletal tissues in various vertebrate species might be compared in an independent way. However, the available data in the literature only allow partial comparative analyses, especially for molecular comparative studies (Table 1).

**TABLE 1**
**Signals Inducing Skeletal Tissues and Ray Dermal Bone Differentiation in Vertebrates**

| Induced Tissues | BMP2 | SHH | RA | IHH |
|---|---|---|---|---|
| Bone | +[23][d2]<br>+[24][d2]<br>+[56][c3] | +[81][d3]<br>+[82][ab2] | +[13][ac3]<br>+[87][ac2] | [24][f1]<br>[32][f3]<br>[97][f2]<br>+[98][e1] |
| Cartilage | +[72][c1]<br>+[73][ac2]<br>+[74][ac2] | +[83][d1]<br>+[84][d2]<br>+[85][c3] | –[13][ac3]<br>–[57][ef1]<br>–[88][c1]<br>+[89][ac1]<br>+[90][ac2] | **[55][fg3]**<br>–[98][e1]<br>–[99][ec1]<br>+[100][ac2]<br>[101][f13]<br>+[102][ac2] |
| Dentine | +[38][ab2]<br>+[76][ac2]<br>[77][f1]<br>[78][f1] | **[9][d1]** | **±[91][cg2]**<br>–[92]**[cg1]**<br>[93]**[cg1]** | [103][f1] |
| Enamel | [77][f1]<br>[78][f1]<br>[79][f1] | **[9][d1]**<br>**+[86][e1]** | **–[92][cg1]**<br>**[93][cg1]**<br>**[94][cg1]** | n.d. |
| Lepidotrichia | +[12][d5]<br>[80][f4,5] | +[12][d5]<br>[80][f4,5] | **+[6][c5]**<br>**–[95][c4]**<br>**–[96][c4]** | [8][f4,5] |

*Note:* a: MSC *in vitro;* b: MSC *in vivo/ex vivo;* c: purified molecules administration; d: gene transfer; e: transgenic/mutant mice; f: *in situ* hybridization; g: explants culture; 1: any skeletal tissue ontogeny; 2: any skeletal tissue renewal from MSCs; 3: any skeletal tissue regeneration; 4: fish lepidotrichia ontogeny; 5: fish lepidotrichia regeneration; +/–: promotion/repression of skeletal formation; **bold:** involved in pattern formation or not essential for differentiation; n.d.: not determined. Shh: sonic hedgehog; RA: retinoic acid; Ihh: Indian hedgehog.

Moreover, when interpreting experimental results, authors might discriminate between pattern formation (previous to tissue differentiation), and cell differentiation/ECM synthesis and mineralization (proper tissue differentiation). In some cases, possible pattern-forming processes will not be easily separated from cell differentiation events (i.e., fracture healing[56]; *in vitro* studies[57]). Some of these topics have been considered in recent reviews[58,59,60].

At the molecular level, orthology among inducing signals and possible signaling modules that induce osteogenesis during vertebrate development have already been proposed[61,62]. Recent studies suggest independent evolution (phenogenetic drift) of some ECM components in enamel and dentine among vertebrate species[10].

Our comparative molecular study will be focused then on these two aspects:

- ECM components
- Signals that induce progenitor cells to differentiate into the main skeletal tissues

## FIN RAY LEPIDOTRICHIA ECM IS DIFFERENT TO ANY OTHER SKELETAL TISSUE IN FISHES

The comparison of the expression of various collagen genes allows us to show that the lepidotrichia differ from other skeletal tissues. *collagen alpha 1* (*type X*) (*col10a1*) chain gene mRNA, as well as *col1a1*[63], *col1a2,* and *col2a1* mRNAs, have been isolated from a subtraction library or by DDRT-PCR from regenerating fins of zebrafish[7]. It has also been shown that *col10a1* is expressed in cartilage, intramembranous and endochondral bone of developing zebrafish larvae, and fin regenerates[8]. *col2a1*, a marker for cartilage in mammalian species, is expressed in the hypochord, mesenchyme of the neurocranium, pharyngeal arches, and the cartilage of developing fins in zebrafish[64,65,66], but not in enameloid and dentine[34]. Zebrafish mutants for *col1a1* show bone defects, suggesting that this gene is required during bone and fin formation, but they do not show any cartilage phenotype[63]. In addition, *col1a1* is expressed in cells surrounding the cartilage, but not in the cartilages themselves[63]. *col1a1* is also expressed in enameloid and dentine in fish teeth[10,34].

In summary, developing and regenerating lepidotrichia express or require *col10a1*[7,8], *col1a2*[7], *col1a1*[7,63], and *col2a1*[7,64]. This combination of genes is not found in any other skeletal tissues of fish. Cartilage formation does not require *col1a1*[63], and *col2a1* is not expressed in bone[65,66] or teeth[34]. All these results are consistent with the hypothesis that fin lepidotrichia could be a special type of skeletal tissue in fish[3].

## BMP2 INDUCES DIFFERENTIATION OF CARTILAGE- AND BONE-FORMING CELLS IN MAMMALS AND OF LEPIDOTRICHIA-FORMING CELLS IN FISH FINS

In 1889, Senn[67] observed that decalcified bone induces healing of bone defects. Urist[68] showed that decalcified bone matrix (DBM) induces ectopic bone formation. Since then, a number of molecules have been found to mediate this effect[69]. The first protein activity was discovered by Sampath and Reddi in 1981[70]. In a review, Reddi describes the general effect of BMPs on osteogenesis induction both *in vivo* and *in vitro*[69]. The first extensive study of BMPs in the search for therapies against human bone disorders was published by Burkus et al.[71]. In this work, more than 600 patients were studied to prove statistically the positive effect of BMP2 in long bone fractures repair, which includes formation of both cartilage and bone. BMP2 shows a positive effect on chondrogenesis and dentine differentiation induction following many different experimental procedures (Table 1)[38,72,73,74,75,76,77].

Finally, Quint et al.[12] showed the involvement of BMP2 in lepidotrichia differentiation. Indeed, the ectopic expression of *bmp2b* in the fin regenerate induced ectopic bone formation, while overexpression of Chordin, a BMP antagonist, impaired bone formation[104] (Table 1).

## ADDITIONAL EXPERIMENTS WITH OTHER INDUCTIVE SIGNALS ALSO SUPPORT THE NOTION THAT LEPIDOTRICHIA IS A SPECIAL TYPE OF SKELETAL TISSUE

A variety of techniques has been used to study the effects of other inducing signals as reported in Table 1: MSC *in vitro* (a)[105], MSC *in vivo/ex vivo* (b)[105,106], purified molecular administration (c)[15,70,88], gene transfer (d)[12,23,24,25,83], and transgenic/mutant mice (e)[9,56]. Their inductive activities have been studied during skeletal tissue ontogeny (1, 4 in Table 1), renewal (*in vitro* or *ex vivo* studies) (2 in Table 1), or regeneration (3, 5 in Table 1) in several vertebrate model systems or humans.

Our proposal of fin regeneration as a model system for skeletal disorders in mammals is based on the data shown in the table and further explanations below.

As an example, the signal *Sonic hedgehog* is able to ectopically induce any skeletal tissue, bone, enamel/enameloid, dentine, cartilage, or lepidotrichia (see Table 1). However, enamel and dentine may differentiate in the absence of *Shh* function in transgenic mice[9]. Absence of *shh* signaling leads to outgrowth arrest during fin regeneration[12] (Table 1), a similar phenotype to the one observed after collagen synthesis inhibition[107]. This is compatible with *shh* affecting lepidotrichia ECM formation during regeneration. This would further suggest, as we have discussed according to its collagen content, that lepidotrichia is not dentine as previously proposed[4] and not enamel either.

During both endochondral and intramembranous ossification, several differentiation events occur. These events can be disclosed using retinoic acid (RA). Retinoic acid is a potent repressor of cartilage formation[13,56,88], whereas it induces both terminal chondrocyte[89,90] and osteoblast differentiation[13,87]. It has also been shown that retinoic acid has stage-specific positive and negative effects on tooth morphogenesis[91]. There is not yet a clear conclusion from lepidotrichia studies, but retinoic acid participates in both ray pattern formation and differentiation (Table 1)[6,95,96].

It has also been shown that *Indian hedgehog (Ihh)* couples chondrogenesis and osteogenesis by repressing hypertrophic chondrocyte formation[98,99] and promoting osteoblast lineage commitment[98]. Moreover, it has a positive effect on late phases of chondrocyte differentiation[100,102]. *Ihh* is also expressed during tooth formation[103]. Studies on zebrafish ontogeny and fin regeneration[8] suggest that *ihh* is expressed in developing and regenerating fins during scleroblast differentiation. However, conclusions must wait for functional analysis (Table 1).

Fibroblast growth factors (FGFs) are able to induce all types of skeletal tissues[108,109,110,111,112, 113,114,115,116,117]. Several arguments further suggest that FGF could be necessary for lepidotrichia formation[118]. The inhibition of FGF signaling pathway stops fin outgrowth[119] and modulation of the FGF signaling regulates the rate of fin outgrowth[120,121]. *Wnt* genes are also involved in the regulation of chondro-osteogenesis differentiation in mammals[122] and *wnt3a, wnt5, and β-catenin* genes are expressed during fin regeneration[48]. TGFβs are other signals inducing chondro-osteogenesis[123] that could be studied in regenerating fins. Although further studies must be carried out, the arguments we have discussed suggest that lepidotrichia behave similar to the main skeletal tissues under experimental studies using inducing factors.

## FUTURE PERSPECTIVES IN FIN REGENERATION STUDIES

From an evolutionary point of view, dermal fin rays are not homologous to the tetrapod limb. Furthermore, morphological and molecular evidence suggests that lepidotrichia is a special type of skeletal tissue different to any main skeletal tissue. Despite this recognized peculiarity of the lepidotrichia, the above-mentioned results suggest a close similarity between the effects of inducing signals on the main skeletal tissues in vertebrates and on ray dermal bone formation. Further experimental data using known chondro-osteogenic inducing signals in mammals could provide further evidence to support this idea. This empirical support occurs above any consideration of homology between tissues. For instance, limb development in arthropods and vertebrates are nonhomologous processes, according to comparative anatomy, but use similar controlling genetic mechanisms[124].

From a medical view, several clinical groups have already obtained sufficient data to initiate bone disorder therapies[17,105,125,126,127,128,129,130,131,132,133,134,135,136,137,138,139,140,141,142, 143]. In order to improve the effect of the inducing signals that are used, a variety of delivery systems are being tested[144,145,146,147]. These materials facilitate the regenerating activity of the cells and increase the efficacy of the treatment. Modifications in the amino acidic sequence of released signals may also be necessary to facilitate stable interactions of the factors with MSCs in culture and *ex vivo* studies[19,20] (Fig. 2). According to all empirical data reviewed above, we propose the use of fin ray regeneration/development studies as a model system for preclinical studies on skeletal tissue disorders.

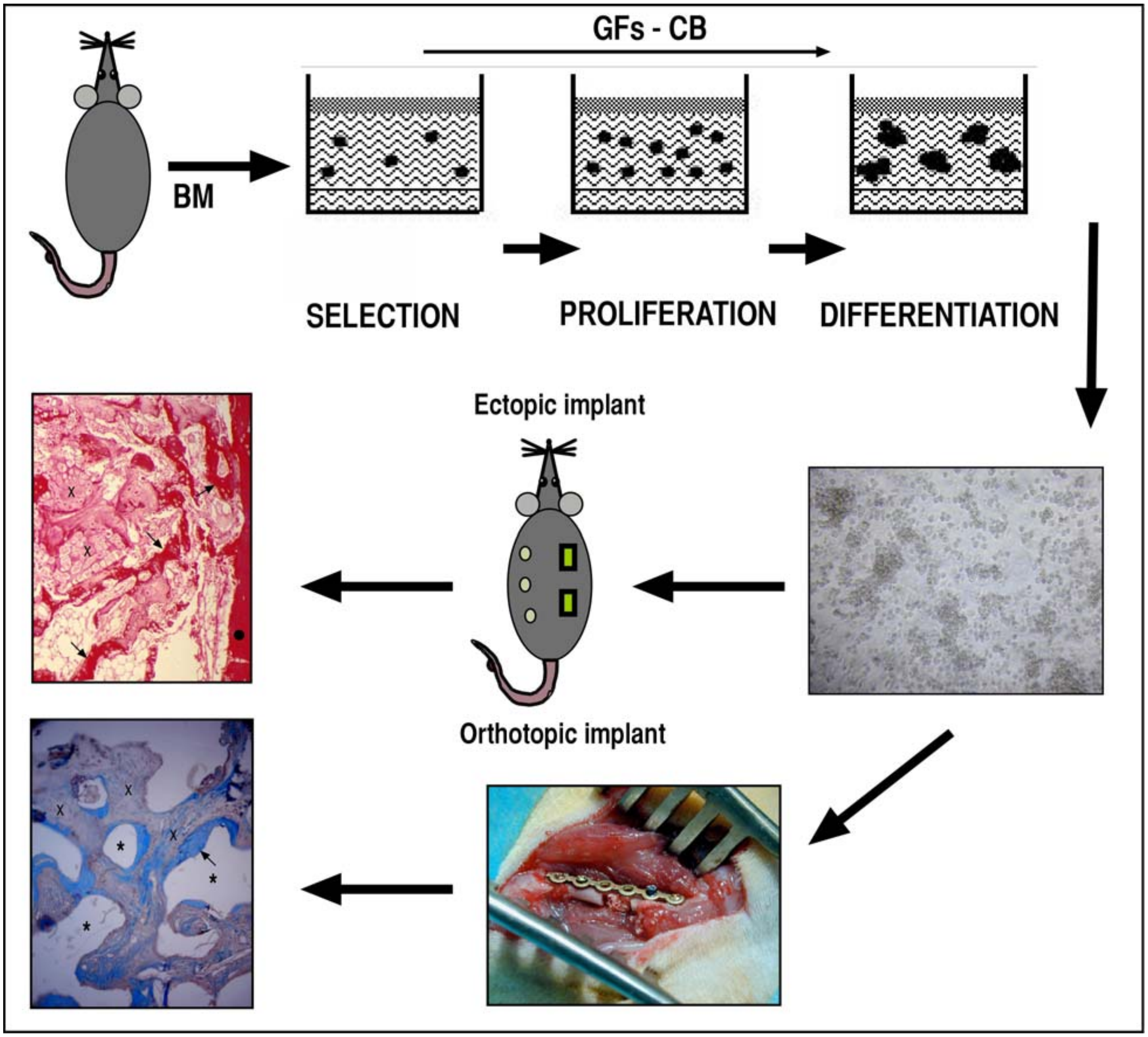

**FIGURE 2**. Culture of bone marrow cells for *in vivo* implantation. Bone marrow cells from rats are cultured in three-dimensional collagen during a short period of time in the presence of 0.5% FBS. After this starvation period, a cell population can be selected. These cells cultured in the presence of 10% FBS proliferate significantly increasing the cell population. Dexamethasone, β-glycerolphosphate, and ascorbic acid are added to induce osteogenic differentiation. All this process can be modulated by TGFβ1 and different BMPs. Cells extracted from the final culture may be ectopically grafted in a DBM chamber. After several weeks, implanted cells produce cartilage and bone inside the chamber. Alternatively, cells from *in vitro* culture can be orthotopically grafted using hydroxyapatite (HA) as biomaterial. After several weeks, a new tissue occupies the center of the HA. In this new tissue, both cartilage and bone can be easily identified. Arrows (bone tissue), X (cartilage tissue), dots (DBM wall), asterisk (trabeculae of HA).

## CONCLUSIONS

For a complete understanding of actinopterygian lepidotrichia nature, it is necessary to complete several studies. However, the experimental data accumulated until now support the notion that ray dermal bone, or lepidotrichia, is actually a special type of skeletal tissue when analyzed at the molecular level. Despite this dissimilarity to other skeletal tissues, these empirical evidence also suggest that studies on lepidotrichia (i.e., regenerating fins) could be of interest in preclinical studies of skeletal tissues disorder therapies. At least three inducing signals or modulators, SHH, BMP-2, and retinoic acid, have been proved to have an outstanding effect on lepidotrichia, and the rest of skeletal tissues in vertebrates. Further studies with other inducing signals could provide light to support our hypothesis of preclinical interest.

## ACKNOWLEDGMENTS

The authors are indebted to *TheScientificWorldJOURNAL* Editor, Marie-Andrée Akimenko, for handling our manuscript. We also acknowledge María Dolores López Ávalos for critical reading of our manuscript. This work has been supported by grants from the Spanish Ministry of Education and Science, BOS2002-00347, from the Spanish Ministry of Health, (PI02/1758, PI06/1855), from Junta de Andalucía, PAI (CVI/217), and from Fundación Progreso y Salud (TC 201.1.2/04), under the management of Fundación IMABIS, Spain. One patent is associated to this study PCT/ES2005/000287[148].
